# Intracellular Communication among Morphogen Signaling Pathways during Vertebrate Body Plan Formation

**DOI:** 10.3390/genes11030341

**Published:** 2020-03-24

**Authors:** Kimiko Takebayashi-Suzuki, Atsushi Suzuki

**Affiliations:** 1 Amphibian Research Center, Hiroshima University, 1-3-1 Kagamiyama, Higashi-Hiroshima, Hiroshima 739-8526, Japan; 2 Graduate School of Integrated Sciences for Life, Amphibian Research Center, Hiroshima University, 1-3-1 Kagamiyama, Higashi-Hiroshima, Hiroshima, 739-8526, Japan

**Keywords:** BMP, FGF, Wnt, retinoic acid, dorsal–ventral and anterior–posterior axis formation

## Abstract

During embryonic development in vertebrates, morphogens play an important role in cell fate determination and morphogenesis. Bone morphogenetic proteins (BMPs) belonging to the transforming growth factor-β (TGF-β) family control the dorsal–ventral (DV) patterning of embryos, whereas other morphogens such as fibroblast growth factor (FGF), Wnt family members, and retinoic acid (RA) regulate the formation of the anterior–posterior (AP) axis. Activation of morphogen signaling results in changes in the expression of target genes including transcription factors that direct cell fate along the body axes. To ensure the correct establishment of the body plan, the processes of DV and AP axis formation must be linked and coordinately regulated by a fine-tuning of morphogen signaling. In this review, we focus on the interplay of various intracellular regulatory mechanisms and discuss how communication among morphogen signaling pathways modulates body axis formation in vertebrate embryos.

## 1. Introduction

A morphogen is defined as a molecule released from a localized source that determines several different cell fates and controls morphogenesis by regulating gene expression in a concentration-dependent manner [1,2]. During embryonic development, most morphogens are secreted molecules that bind to transmembrane receptors, activate intracellular signal transducers, and then regulate the expression of downstream target genes. Well-known morphogens are bone morphogenetic proteins (BMPs), Nodals, and Activins, which all belong to the transforming growth factor-β (TGF-β) family, fibroblast growth factors (FGFs), and Wnt family proteins [3,4,5,6,7,8,9,10,11,12,13,14,15]. There are a few exceptions to the typical morphogen: retinoic acid (RA), a small compound synthesized from vitamin A (all-trans-retinol), works as a morphogen in embryos [16,17,18,19], and Bicoid functions as a morphogen transcription factor in the syncytial blastoderm of *Drosophila* embryo that contains many nuclei in a large cytoplasm [20].

**Figure 1:**
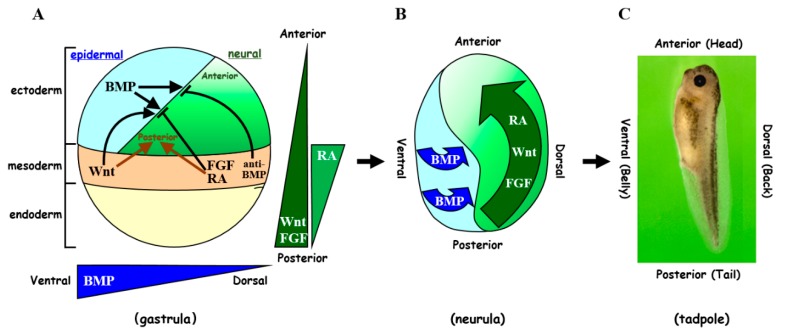
Cell fate specification by morphogen signaling during body axis formation in *Xenopus* embryos. (**A**) At the gastrula stage, bone morphogenetic protein (BMP) and Wnt ligands promote the epidermal fate of the ectoderm on the ventral side. Neural tissue is formed from the ectoderm when BMPs are inhibited by BMP antagonists (anti-BMP; Noggin, Chordin, and Follistatin) emanating from the dorsal mesoderm (Spemann’s organizer), which later becomes the notochord. (**B**) By the neurula stage, the induced neural tissue is regionalized along the anterior-posterior (AP) axis by the posteriorizing factors fibroblast growth factor (FGF), Wnt, and retinoic acid (RA), and the neural plate above the notochord forms the neural tube which will develop into the brain and spinal cord. (**C**) By the tadpole stage, a variety of organs and tissues such as brain, eyes, somites, and tail are formed along the dorsal–ventral (DV) (back–belly) and AP (head–tail) axes. Green, neural/dorsal ectoderm; blue, epidermal/ventral ectoderm; orange, mesoderm (marginal zone); yellow, endoderm.

In early *Xenopus* embryos, the regulation of body axis formation by morphogens has been thoroughly investigated, and it has been shown that a gradient of BMP signaling determines the dorsal–ventral (DV) axis (Figure 1). During gastrulation, ventral ectodermal cells with high BMP signaling acquire an epidermal fate; however, ectodermal cells close to the dorsal marginal zone (Spemann’s organizer), where genes for BMP antagonists (*noggin*, *chordin*, and *follistatin*) are expressed, cannot receive BMP signaling and adopt a neural fate [4,14,15,21,22,23,24,25]. BMP ligands bind to two different transmembrane serine/threonine kinase receptors (type I and type II) and activate cellular responses to induce biological functions [6,7,26,27,28,29,30,31,32]. After BMP ligand binding, the type II receptor forms a complex with a type I receptor and phosphorylates/activates the type I receptor, leading to the subsequent C-terminal phosphorylation of the signal transducer Smad1/5/8 in the cytosol. The C-terminally phosphorylated Smad1/5/8 (pSmad1/5/8) oligomerizes with Smad4 and then translocates into the nucleus. In the nucleus, a complex of pSmad1/5/8 and Smad4 interacts with other accessory molecules and functions as a transcriptional activator or repressor. A high level of BMP signaling induces the expression of the BMP downstream target genes *ap-2 (tfap2a)*, *dlx3/5*, *vent-2 (ventx2.2)*, and *msx1* on the ventral side of gastrula embryos and downregulates the expression of neural marker genes such as *sox2* and *ncam* [33,34,35,36,37,38]. As a result, BMPs determine the epidermal/ventral fate while suppressing the neural/dorsal fate and regulate the DV axis of embryos. 

The anterior-posterior (AP) patterning of embryos is regulated by FGF, Wnt, and RA signaling (Figure 1) [8,39,40,41,42]. FGF signaling is transduced by tyrosine kinase receptors and activates the mitogen-activated kinase (MAPK) pathway consisting of MAPKKKs (Ras and Raf), MAPKKs, and MAPKs (also called MEKs and ERKs, respectively) [12,13]. FGF4 induces the expression of homeobox genes, such as *hoxa7, hoxb9, hoxc6*, and *caudal*-related genes, that have important roles in the patterning of the AP axis; some of these genes have also a role in suppressing anterior development [43,44,45,46,47]. Wnt ligands form a complex with the multi-pass transmembrane receptor Frizzled and the Wnt co-receptors of the low-density lipoprotein (LDL) receptor-related protein (Lrp) family, specifically Lrp5 and Lrp6, and this interaction results in the stabilization of β-Catenin in the cytoplasm [48,49,50,51,52]. Accumulated β-Catenin translocates into the nucleus and induces the expression of Wnt target genes. In the absence of Wnt ligands, glycogen synthase kinase-3β (GSK-3β) participates in the destruction complex composed of adenoma polyposis coli (APC), Axin, β-Catenin, and the E3 ubiquitin ligase β-TrCP. The formation of the destruction complex leads to β-Catenin ubiquitination and degradation via the proteasome pathway. Wnt/β-Catenin signaling induces the expression of the homeobox genes *cad3* and *meis3* that are important for posterior development [53,54]. RA interacts with nuclear RA receptors (RARs) or retinoid X receptors (RXRs), and RARs and/or RXRs bind to an RA response element in the regulatory region of target genes [19,55,56]. RA signaling controls the expression of *hoxa1, hoxb1, hoxa3, hoxd4*, and *vhnf1* that pattern the posterior part of the brain [17,57,58]. It has been shown that FGF, Wnt, and RA signaling cascades function in concert to regulate gene expression along the AP axis of the embryo [12,59,60,61,62,63]. 

To ensure the correct organization of the body plan, the processes of DV and AP axis formation must be linked and coordinately regulated by the fine-tuning of morphogen signaling. In the following parts of this review, we discuss how communication among morphogen signaling pathways, especially BMP, FGF, Wnt, and RA signaling, is achieved intracellularly and functions as the molecular link that coordinates DV and AP patterning during body plan formation in vertebrates.

## 2. Phosphorylation of Smad

**Figure 2:**
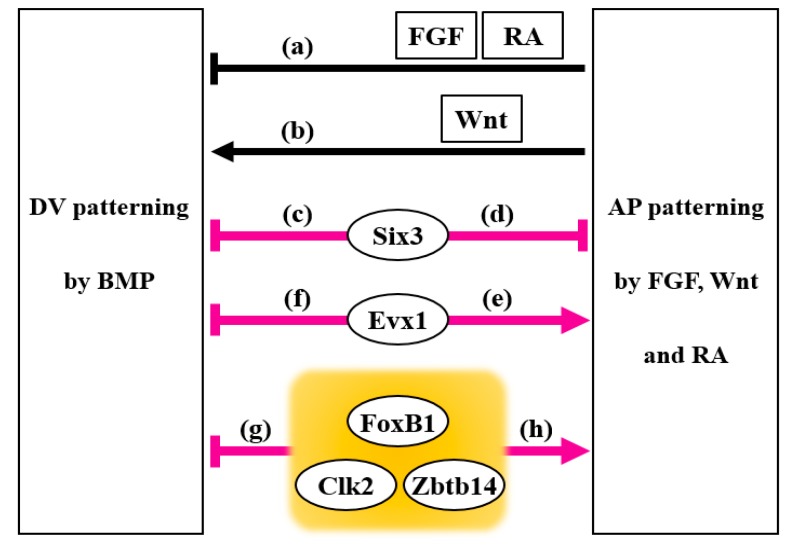
Interconnected regulatory pathways in the control of DV and AP axis formation. BMP signaling determines the DV axis by inducing ventral fate, while the AP patterning is controlled by the FGF, Wnt, and RA signaling pathways. (**a**) FGF and RA inhibit BMP signaling by promoting Smad1/5/8 degradation. On the other hand, (**b**) Wnt stimulation enhances BMP signaling by the stabilization of C-terminally phosphorylated Smad1/5/8. Six homeobox 3 (Six3) inhibits both BMP (**c**) and Wnt pathways (**d**) by suppressing the expression of BMP and Wnt ligands, respectively. (**e**) Even-skipped homeobox 1 (Evx1) induces the expression of RA-synthesizing enzyme and suppresses the expression of RA-degrading enzyme to activate RA signaling. (**f**) Evx1 also interferes with the BMP pathway by suppressing BMP ligand expression. (**g**) Forkhead box B1 (FoxB1), Zinc-finger and BTB/POZ (Broad-complex, Tramtrack, and Bric-a-brac/Poxvirus and Zinc-finger) domain-containing protein 14 (Zbtb14), and Cdc2-like kinase 2 (Clk2) (highlighted in the orange square) inhibit BMP signaling by reducing the level of C-terminally phosphorylated Smad1/5/8, and (**h**) these factors enhance Wnt and/or FGF signaling through different mechanisms as shown in Figure 3.

Several intracellular factors have been shown to function as molecular links between morphogen signaling pathways that coordinate DV and AP patterning in the embryo. A well-studied intracellular factor is Smad1, which primarily transduces BMP signaling. Smad1 has a structure consisting of three domains: Mad-homology 1 (MH1), MH2, and a linker region between the MH1 and MH2 domains [30,64]. The phosphorylation status of selected sites on Smad1 positively or negatively regulates its activity. MAPK, which is activated by epidermal growth factor (EGF) through a tyrosine kinase receptor, phosphorylates the linker region of Smad1, and this phosphorylation inhibits the nuclear accumulation of Smad1 in the mink lung epithelial cell line [65]. During *Xenopus* embryogenesis, FGF8 and insulin-like growth factor 2 (IGF2) promote neural induction (dorsalization) by inhibition of BMP signaling via MAPK-mediated Smad1 linker phosphorylation (inhibition path (a) in Figure 2) [66,67]. FGF/IGF signaling causes neural induction; however, Wnt signaling enhances epidermal differentiation (ventralization) of chick epiblast cells, and inhibition of Wnt signaling by a soluble fragment of Frizzled protein promotes neural induction [68]. In addition, the Wnt antagonist Dickkopf-1 induces the differentiation of anterior neural tissue in *Xenopus* and zebrafish embryos [69,70], further supporting the proposed roles of Wnt signaling in epidermal differentiation and inhibition of neural induction. 

**Figure 3:**
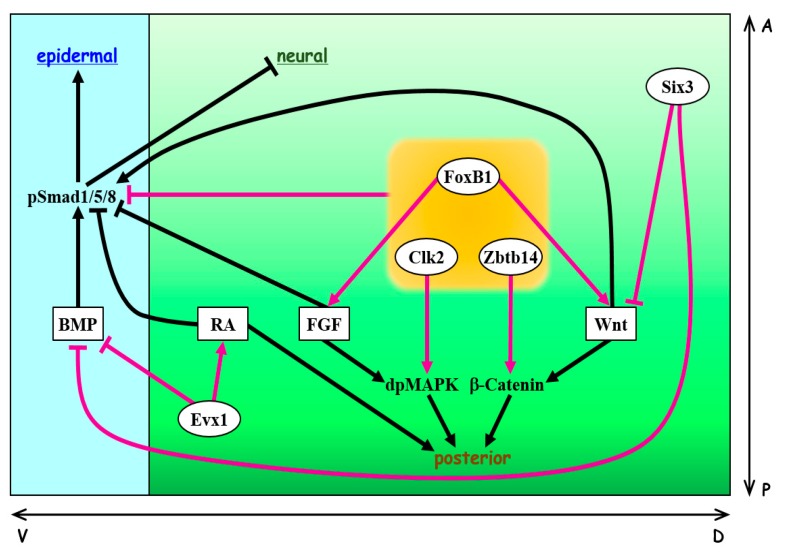
Intracellular regulators that link morphogen signaling pathways during DV and AP patterning of the ectoderm in vertebrate embryos. The genes *six3* and *evx1* are expressed in the anterior and posterior neuroectoderm, respectively. Although both Six3 and Evx1 interfere with the BMP pathway by suppressing BMP ligand expression, these factors have opposite functions in AP patterning of the ectoderm. Six3 suppresses the expression of Wnt ligands to facilitate the formation of anterior neural tissue. Evx1 activates RA signaling by the induction of RA-synthesizing enzyme and the suppression of RA-degrading enzyme and thus enhances posterior development. FoxB1, Zbtb14, and Clk2 (highlighted in the orange square) reduce the level of C-terminally phosphorylated Smad1/5/8 (pSmad1/5/8) to inhibit BMP signaling, thereby promoting neural induction (dorsalization) of the ectoderm. These three factors are also involved in the posteriorization of neural tissue induced by BMP inhibition, albeit through different mechanisms. FoxB1 induces the expression of Wnt and FGF ligands, and Zbtb14 enhances Wnt signaling by increasing the accumulation of β-Catenin. Clk2 elevates the level of diphosphorylated mitogen-activated kinase (dpMAPK) induced by FGF, and thus Clk2 promotes FGF-mediated posteriorization. Green, neural/dorsal ectoderm; blue, epidermal/ventral ectoderm; D, dorsal; V, ventral; A, anterior; P, posterior.

The molecular mechanism linking Smad1 phosphorylation and BMP, FGF, and Wnt signaling has been identified [27,28,32,71,72,73]. After the phosphorylation of two C-terminal serine residues in Smad1 by a BMP type I receptor, the PXSP motifs of the Smad1 linker region are phosphorylated by MAPK that has been activated by FGF signaling. GSK-3β then phosphorylates the serine or threonine residues (S/TXXXS motifs) that are located four amino acids upstream of the MAPK phosphorylation sites in the Smad1 linker region. Smad1 linker phosphorylation enables the E3 ubiquitin ligases Smurf1 and Smurf2 to interact with Smad1; this interaction is followed by polyubiquitination and degradation of Smad1 via the proteasome pathway [31,71,72,73,74,75,76]. As GSK-3β is inactivated by Wnt signaling, Wnt stimulation causes a more prolonged stabilization of C-terminally phosphorylated Smad1 (pSmad1) [73]. This suggests that Wnt signaling enhances epidermal differentiation (ventralization) by extending the duration of BMP signaling (activation path (b) in Figure 2). It has been proposed that not only the strength of morphogen signaling but also its timing and duration are crucial for responding cells to interpret extracellular stimulation [1,77,78]. Indeed, different durations of BMP exposure cause different levels of intracellular signaling activity that induce distinct dorsal neuronal subtypes in the chick neural tube [79] and in mouse and human embryoid bodies cultured *in vitro* [80]. This finding explains how a limited number of morphogens can be effectively utilized to induce various cell types during development. Since BMP and Wnt ligands are co-expressed in some cell types during early development and organogenesis, Wnt signaling may modulate BMP signaling by affecting the duration of BMP signaling through the phosphorylation of the Smad1 linker region. Intriguingly, RA enhances MAPK-mediated Smad1 linker phosphorylation by inducing the expression of the MAPK activator *gadd45* [81]. Moreover, RA signaling promotes the interaction between pSmad1 and its E3 ubiquitin ligase Smurf2, followed by ubiquitination and degradation of pSmad1. Therefore, the posteriorizing factor RA may also inhibit BMP signaling by regulating the duration of BMP signaling during neural development (inhibition path (a) in Figure 2). In *Drosophila*, it has been reported that Mad (the *Drosophila* homolog of Smad1) is used in both BMP and Wnt signaling pathways [82]. While the BMP receptor Thickveins phosphorylates the C-terminus of Mad to activate BMP signaling, unphosphorylated Mad is required for canonical Wnt signaling, and thus the utilization of Mad in BMP signaling prevents the transduction of Wnt signaling. Although not confirmed in vertebrate embryos, this novel mechanism adds another layer of crosstalk between BMP and Wnt signaling pathways. These observations suggest that to ensure the correct establishment of the body plan, BMP, FGF, Wnt, and RA signaling pathways are tightly linked and coordinately regulated at the level of Smad1/5/8 phosphorylation (Figure 3). 

In zebrafish, Smad1 and Smad8 (also known as Smad9) have a redundant function in the DV patterning of embryos [83]; in the chick, Smad1 and Smad5 are largely interchangeable for dorsal spinal cord neurogenesis [84]. In the mouse, embryos that are null for *smad1* or *smad5* die at E9.5–E11.5; however, embryos that are null for *smad8* survive and develop normally [85,86,87]. Moreover, although *smad1*^+/-^ and *smad5*^+/-^ heterozygous mice are viable and fertile, *smad1*^+/-^; *smad5*^+/-^ double-mutant embryos die around E10.5 [87], suggesting that the functions of mouse Smad1/5/8 are distinct but partially overlap. This might explain the fact that *smad1* mutant mice lacking the MAPK phosphorylation site in the linker region show defects in gastric epithelial homeostasis but otherwise show normal early development [88]. Therefore, compared to other vertebrate models, the role of Smad1/5/8 in body axis formation in mouse development is less well documented due to its functional redundancy and the embryonic lethality of mutants. In future, it will be necessary to determine whether mammalian Smad1/5/8 plays an important role in body axis formation through the integration of morphogen signaling pathways.

## 3. Six3 

The gene *six3* (*six homeobox 3*), a vertebrate homolog of the *Drosophila sine oculis* gene [89], plays an important role in craniofacial and brain development [90,91,92,93]. It has been shown that *six3* mutant mice show abnormal craniofacial morphogenesis and lack eyes, nose, and most head structures anterior to the midbrain [94,95]. In zebrafish and *Xenopus*, Six3 represses *bmp4* expression (inhibition path (c) in Figure 2; Figure 3) and vice versa, indicating a mutual antagonism between Six3 and BMP signaling [96]. Overexpression of Six3 expands the anterior neural plate and promotes cell proliferation. Moreover, exogenous Six3 can rescue the reduction of anterior neural structures caused by a loss-of-function mutation in *chordin*. Thus, Six3 maintains and refines the size of anterior neural tissue by protection against the ventralizing activity of BMPs. Although Wnt signaling is capable of inhibiting *six3* expression, Six3 can repress the expression of *wnt1* and *wnt3* in the anterior neuroectoderm that is fated to become the forebrain during mouse and chick development (inhibition path (d) in Figure 2; Figure 3) [94,97]. Mice with a knockout of *fgf8* show expanded expression of *six3* toward the posterior ectoderm and fail to form a neural tube [98]. In accordance with this observation, expression of *six3* is limited to rostral neural tissue by FGF signaling in mouse embryonic stem cell aggregates that intrinsically develop a rostral–caudal neural pattern [99]. These findings suggest that the spatially restricted expression of *six3* by Wnt and FGF signaling is necessary to achieve the correct patterning of the ectoderm along the AP axis. Thus, by integrating BMP, Wnt, and FGF signaling, Six3 functions as a key molecule that regulates DV and AP patterning of the ectoderm.

## 4. Evx1 

The transcription factor *even-skipped homeobox 1* (*evx1*, also known as *eve1* or *xhox3*) is expressed in the posterior regions of mouse and *Xenopus* embryos during gastrulation [100,101,102,103]. In zebrafish and *Xenopus*, Evx1 overexpression causes anterior truncation and the induction of posterior marker genes during early development [103,104,105,106]. Consistent with this finding, loss-of-function of Evx1 in zebrafish and *Xenopus* embryos results in the reduced expression of posterior markers and a failure of trunk/tail development [105,107]. Similarly, Evx1 knockdown in human ES cells causes a reduction in the expression of posterior markers and promotes anterior streak and endodermal fates [108]. Although body axis patterning of *evx1*-null mice needs to be analyzed, conditional mutation of *evx1* affects commissural axon projections in the developing spinal cord [109]. 

Zebrafish Evx1 induces the expression of *aldehyde dehydrogenase 1 family member A2/raldh2* (*aldh1a2*), which synthesizes RA from its precursor [105]. As RA acts as an essential morphogen for embryonic axis formation, limb development, and organogenesis, the level of RA needs to be regulated precisely by synthesizing and degrading enzymes [18,19,56]. In addition to the induction of *aldh1a2*, Evx1 suppresses the expression of the RA-degrading enzyme *cytochrome P450 26* (*cyp26*) to activate RA signaling and further promote posterior development [105]. FGF and Wnt signaling suppress the expression of *cyp26* [61,110] and upregulate the expression of *evx1* [105,111,112,113,114]. Thus, together with FGF and Wnt signaling, Evx1 plays an important role in a regulatory network that induces posteriorization by RA signaling (activation path (e) in Figure 2; Figure 3). Evx1 not only promotes posterior development but also enhances neural induction (dorsalization) by suppressing BMP ligand expression (inhibition path (f) in Figure 2; Figure 3) [105]. Accordingly, in the presence of excess BMP ligands, Evx1 is not able to induce the expression of the neural marker genes *sox3* and *hoxb1* [105]. Hence, Evx1 is involved in both posterior development and DV patterning of trunk/tail tissue by connecting RA, FGF, Wnt, and BMP signaling pathways. Although both Six3 and Evx1 dorsalize the embryo by interfering with the BMP pathway, these transcription factors have opposite functions in the regulation of AP patterning, suggesting the presence of transcriptional network hubs controlled by Six3 and Evx1 for the specification of anterior and posterior regions, respectively. 

## 5. FoxB1 

Forkhead box B1 (FoxB1; previously referred to as TWH, Mf3, or Fkh5) is a member of the forkhead box (Fox) transcription factor family and contains a characteristic DNA-binding domain with a winged helix motif [115,116]. Mice deficient in FoxB1 show open neural tube defects, impaired hypothalamus development, and reduced posterior tissue formation [117,118,119,120,121,122]. Expression of *Xenopus foxb1* is detected in the posterior dorsal ectoderm of early gastrula embryos and, at later stages, in the mid- and hind-brain and spinal cord [123]. The expression of *foxb1* is induced by the posteriorizing factors FGF and Wnt. Moreover, we found that *Xenopus foxb1* acts as a downstream gene of Oct25 (Pou5f3.2) that inhibits BMP responses; FoxB1 also promotes neural induction at the expense of epidermal differentiation [124]. Overexpression of FoxB1 inhibits BMP-dependent epidermal differentiation by reducing the levels of pSmad1/5/8 in *Xenopus* ectodermal cells. Upon BMP stimulation, pSmad1/5/8 translocates into the nucleus and undergoes dephosphorylation of its C-terminal sites by protein phosphatases, followed by recycling via nucleocytoplasmic shuttling [27,31,125,126]. FoxB1 is localized in the nucleus and interacts preferentially with the unphosphorylated form of Smad8, thereby sequestering Smad8 in the nucleus [124]. Through this mechanism, FoxB1 reduces the levels of cytoplasmic Smad8 available for phosphorylation/activation by BMP receptors and thus suppresses BMP signaling to promote neural/dorsal fate of the ectoderm (inhibition path (g) in Figure 2; Figure 3). 

Knockdown of FoxB1 in *Xenopus* showed that FoxB1 is required for the formation of posterior neural tissue and the suppression of anterior development [124]. FoxB1 upregulates the expression of Wnt and FGF ligand genes (*wnt8*, *fgf3*, and *fgf8*); overexpression of these genes can rescue AP patterning defects in FoxB1-knockdown embryos (activation path (h) in Figure 2; Figure 3). Therefore, FoxB1 regulates both DV and AP patterning of the ectoderm during early *Xenopus* embryogenesis through the regulation of Wnt and FGF signaling pathways. Although the inhibition of endogenous FoxB1 function does not cause significant defects in DV patterning, the double knockdown of FoxB1 and Oct25 results in a severe reduction in the expression of the neural marker *sox2* and causes the expansion of *epidermal keratin* (*keratin 12*, *gene 4*; *xk81*) expression into neural plate territory. FoxB1 functions both downstream of and in concert with Oct25; therefore, FoxB1 forms a feed-forward network with Oct25 which is important for induction and/or maintenance of neural tissue. In summary, FoxB1 controls the establishment of the DV and AP axes of the ectoderm by modulating BMP, Wnt, and FGF signaling.

## 6. Zbtb14 

Zbtb14 (previously called ZF5, ZNF478 or ZFP161) is a zinc-finger and BTB/POZ (Broad-complex, Tramtrack, and Bric-a-brac/Poxvirus and Zinc-finger) domain-containing protein [127,128,129,130], and *Xenopus* Zbtb14 promotes neural tissue formation at the expense of epidermis in early embryos [131]. Similarly to FoxB1, overexpression of Zbtb14 induces posterior neural tissue in the ectoderm. Moreover, Zbtb14 is required for the formation of posterior neural tissues and the suppression of anterior neural development, thus controlling both DV and AP patterning of the ectoderm. Zbtb14 reduces the levels of Smad1/5/8 and pSmad1/5/8, thereby suppressing BMP signaling (inhibition path (g) in Figure 2; Figure 3). The reduction of pSmad1/5/8 requires the ubiquitin–proteasome pathway, and Zbtb14 interacts with the inhibitory Smads (Smad6 and Smad7) and the Smad ubiquitin ligase Smurfs. It is therefore likely that Zbtb14 acts through ubiquitin-mediated degradation of Smad1/5/8. Furthermore, Zbtb14 increases Wnt signaling by promoting the accumulation of β-Catenin through interaction with β-TrCP, which targets β-Catenin for ubiquitination and proteosomal degradation (activation path (h) in Figure 2; Figure 3). The BTB/POZ domain is known to enhance protein–protein interactions, and some Zbtb proteins function as substrate-specific adaptors by binding to the E3 ubiquitin ligase Cullin3 via the BTB/POZ domain [132,133,134,135,136,137]. Thus, it is possible that Zbtb14 mediates the interactions of Smad1/5/8 and β-Catenin with the E3 ubiquitin ligases Smurfs and β-TrCP to regulate the ubiquitination status of the signal transducers, resulting in the modulation of the balance between BMP and Wnt signaling. The available evidence indicates that Zbtb14 plays an essential role in the formation of the DV and AP axes by regulating both BMP and Wnt signaling pathways during early *Xenopus* embryogenesis. Intriguingly, mice expressing a C-terminally truncated form of Zbtb14 show severe defects in heart, kidney, and brain organogenesis [138,139]; further analyses of *zbtb14* knockout mice are needed to clarify the role of Zbtb14 in body axis formation in mammals. As dysregulation of BMP and Wnt signaling components also leads to malformations in heart, kidney, and brain [140,141,142,143,144,145,146,147], the phenotypes of Zbtb14 mutant mice may be due, at least in part, to an imbalance of BMP and Wnt signaling. 

## 7. Clk2

We recently reported that Cdc2-like kinase 2 (Clk2) promotes early neural development and inhibits epidermal differentiation in *Xenopus* embryos [148]. Clk2 is a dual-specificity kinase that phosphorylates serine, threonine, and tyrosine residues [149]; it has been shown that Clk2 functions in various biological events including gluconeogenesis, alternative RNA splicing, and cell proliferation [150,151,152]. *Xenopus clk2* is expressed in neural tissues along the AP axis during early embryogenesis [148]. Overexpression of Clk2 increases the expression of both anterior and posterior neural marker genes. Consistently, the expression of *epidermal keratin* is also reduced in embryos overexpressing Clk2, and this suggests that Clk2 promotes dorsalization/neural induction. Clk2 interferes with BMP signaling downstream of BMP receptor activation, and the neural-inducing ability of Clk2 is enhanced by both BMP inhibition and activation of FGF signaling. Mechanistically, Clk2 downregulates the level of pSmad1/5/8 in cooperation with BMP inhibition and increases the level of activated (diphosphorylated) MAPK induced by FGF signaling (inhibition path (g) and activation path (h) in Figure 2, respectively; Figure 3). These findings suggest that Clk2 is involved in the establishment of the DV and AP axes via modulation of the BMP and FGF signaling pathways. Interestingly, the amount of Clk2 protein is increased in the Shank3-deficient autism spectrum disorder (ASD) mouse model [153]. The chemical inhibition of Clk2 restores impaired social motivation in these mice, indicating that *clk2* is one of the causative genes of ASD and is therefore a potential therapeutic target. In addition, abnormal brain outgrowth has been observed in ASD patients [154]. In *Xenopus,* overexpression of Clk2 expands the neural plate by regulating BMP and FGF signaling during early development [148]. Thus, the modulation of BMP and FGF signaling pathways by Clk2 during neural development could have implications for understanding the pathogenesis and future treatment of ASD.

The Clk family consists of four paralogs (Clk1–4). The paralog *clk1* is expressed in the mammalian brain and induces neuronal differentiation of PC12 cells [155]. Moreover, Clk1, Clk2 and Clk4 act in concert with each other in cell division [156]. Therefore, Clk2 and other members of the Clk family may function redundantly during neural development. Mice with a liver-specific conditional knockout of the *clk2* gene show hepatic lipid accumulation when fed a high-fat diet [157]; however, the early embryonic phenotype of *clk2*-null mice has not yet been reported. In future studies, combinatorial inhibition of Clk family members is needed to clarify the role of Clk in body axis formation. 

## 8. Conclusion and Perspectives

In early vertebrate embryos, BMPs determine the DV axis by inducing ventral fate, and the AP patterning is regulated by FGF, Wnt, and RA signaling pathways. In this review, we focused on the molecular links that coordinately regulate the processes of DV and AP axis formation through the fine-tuning of morphogen signaling. Recent advances have revealed an increasing number of intracellular molecules that are important for the integration and balancing of morphogen signaling pathways (Figure 2; Figure 3). Since gene mutations have been found in the components of morphogen signaling pathways in some severe human diseases, it is crucial to study the molecular mechanisms of integrated communication among these signaling pathways to understand the causes of disease. These efforts will lead to the development of animal disease models and potential future therapies. The challenge for future research is to provide a better understanding of how multiple morphogen signaling pathways are able to govern the formation of the body plan in a spatiotemporal fashion through the utilization of an intricate communication system. It has been reported that while initial cell fate specification occurs in a spatially random manner in response to crude morphogen gradients, differentiated cells are organized into sharply segregated domains by cell migration and rearrangements during neural tube formation [158]. Moreover, it has been recently shown in zebrafish that, in a noisy morphogen gradient, cells with unfit signaling values are removed to ensure a robust patterning of the AP axis [159]. Thus, in addition to a further elucidation of the intracellular communications between morphogen signaling pathways, more detailed studies on cell migration and spatial arrangement/dynamics of cells will be required to understand completely the molecular basis of coordinated DV and AP patterning. The application of new techniques, such as real-time quantitative imaging with spatiotemporal resolution at the subcellular level, offers new approaches to the exploration of when and where intracellular signal transducers are activated/inactivated and/or localized in the coordination of morphogen signaling during early development.
